# Effects of Pelvic-Floor Muscle Training in Patients with Pelvic Organ Prolapse Approached with Surgery vs. Conservative Treatment: A Systematic Review

**DOI:** 10.3390/jpm12050806

**Published:** 2022-05-17

**Authors:** Andrea Espiño-Albela, Carla Castaño-García, Esther Díaz-Mohedo, Alfonso Javier Ibáñez-Vera

**Affiliations:** 1 Clinic Alenda Fisioterapia y Osteopatía, Rúa Menéndez y Pelayo, 4, 1ºF, 15005 A Coruña, Spain; andreaesal@hotmail.com; 2 Clinic Fisio-Especialistas, Calle Lope de Rueda, 17, 1ºC, 28009 Madrid, Spain; carla.castanho97@gmail.com; 3 Deparment of Physiotherapy, Faculty of Health Sciences, University of Malaga, Campus Teatinos, 28078 Malaga, Spain; 4 Department of Health Sciences, Faculty of Health Sciences, University of Jaen, Campus las Lagunillas, 23071 Jaen, Spain; ajibanez@ujaen.es

**Keywords:** pelvic organ prolapse, pelvic-floor muscle training, physiotherapy, surgery, conservative treatment, systematic review

## Abstract

The aim of this systematic review was to explore the effectiveness of pelvic-floor muscle training (PFMT) in the treatment of women with pelvic organ prolapse (POP) who had undergone either surgery or only conservative treatment, based on a selection of randomized clinical trials (RCT). The search was carried out in PubMed, Cochrane, Scopus, CINAHL, and PEDro databases between April 2021 and October 2021 using the following MeSH terms or keywords: “pelvic organ prolapse”, “POP”, “pelvic floor muscle training”, “pelvic floor muscle exercise”, “kegel exercise”, and “surgery”. The methodological quality of the studies was assessed using the PEDro scale. Eighteen RCTs were included in this review. The findings showed improvements in symptoms associated with POP, in pelvic-floor function, and in quality of life in women who performed a PFMT protocol. However, PFMT did not produce significant changes in sexual function, and the results of the change in POP stage were inconclusive. When viewing PFMT as a complementary treatment to surgery, no significant improvements were observed in any of the analyzed variables. In conclusion, a PFMT program is an effective way to improve the pelvic, urinary, and intestinal symptoms associated with POP; function of the pelvic floor; and quality of life. PFMT as an adjunct to surgery does not seem to provide a greater benefit than surgical treatment alone. RCTs of higher methodological quality, with a larger sample size and a longer follow-up, are needed to confirm the results.

## 1. Introduction

Pelvic organ prolapse (POP) is defined as the descent of one or more of the pelvic organs (bladder, uterus, and/or rectum) through the walls of the vagina [1,2]. There are different POPs according to the vaginal compartment in which the descent occurs: prolapse of the anterior vaginal wall (cystocele, urethrocele), prolapse of the posterior vaginal wall (enterocele, rectocele), and apical prolapse (uterine prolapse or prolapse of the vaginal vault after a total hysterectomy) [1,2,3,4]. To determine the severity of POP and its evolution, it is recommended to use the Pelvic Organ Prolapse Quantification (POP-Q) system, which is based on the measurement of the distances between six points located in the vagina and the hymen and which classifies the POP in five stages [3,5,6].

The cause of POP is multifactorial, with pregnancy and childbirth being the most frequently associated risk factors [1,3,7]. POP is a common condition, the prevalence of which increases with age, with an estimated 50% of women suffering from POP [1,3,6,8,9,10], although only between 3 and 12% of women report symptoms [2,3,7,8]. Symptoms include a feeling of vaginal heaviness, a sensation of a lump descending into the vagina, a feeling of pelvic discomfort/pressure, and back pain. They are also frequently associated with urinary and intestinal symptoms and sexual dysfunction [4,9], which greatly affect the quality of life of women with POP [2].

For a correct diagnosis of POP, it is necessary to take a detailed history and a complete physical examination. Sometimes additional tests are recommended, such as an evaluation of the post-void residual, a gynecological ultrasound, and/or a urodynamic study [11].

The treatment of POP can be conservative or surgical, depending on the severity of the prolapse and its symptoms, the quality of life of the patient, her general state of health, and her preferences. Conservative treatment includes pelvic-floor muscle training (PFMT), lifestyle changes, pessaries, and estrogen treatment, all aimed at preventing the symptoms for and worsening of POP. Obliterative or reconstructive techniques can be used in surgical treatment, but since treatment does not always involve definitive surgery and symptoms can re-develop [3,6,8,9,10,11], complementary approaches must be considered. Conservative treatment has the advantage of causing fewer adverse effects and complications [12]. The available evidence suggests that PFMT has a positive effect on prolapse symptoms and severity [12,13,14]. For this reason and due to its safety, PFMT is indicated as a first-choice treatment for women with POP [13]. PFMT aims to increase the structural support of the pelvic organs by improving the strength, endurance, and coordination of the pelvic-floor muscles. It can be combined with other conservative treatments and can also be applied both before and after surgery [12,13]. Despite this, there are no studies considering whether the observed effects of PFMT are the same in women who have undergone POP surgery compared with women who have undergone conservative treatment. PFMT is usually studied in patients under conservative treatment; nonetheless, there is a high interest of analyzing both approaches in order to plan the best personalized treatment in each case.

For all the above, the main objective of our review was to obtain updated evidence on the efficacy of PFMT in the symptoms associated with POP (pelvic, urinary, and intestinal), in the POP stage, in the function of the pelvic floor (strength and endurance), and in the quality of life and sexual function of women with POP who have undergone either surgery or only conservative treatment.

## 2. Materials and Methods

### 2.1. Study Design

A systematic review based on the recommendations of the Preferred Reporting Items for Systematic Reviews and Meta-Analyses (PRISMA) (2020 guideline version) was carried out. The PROSPERO register was checked to ensure similar updated reviews did not exist.

### 2.2. Search Strategy

The electronic databases consulted for the relevant published literature were PubMed, Scopus, Cumulative Index to Nursing and Allied Health Literature (CINAHL), the Cochrane Library, and Physiotherapy Evidence Database (PEDro). In order to identify as many articles as possible, several searches were carried out between April 2021 and October 2021, with the last search being conducted on 17 October 2021. The search strategy was developed based on the PICOS system: patient (patients with POP), intervention (pelvic-floor muscle training), comparison (comparison of PFMT with usual care or exercise without PFMT or surgery), results (prolapse stage in POP-Q system, patient-reported symptoms, quality life, sexual function, and pelvic-floor function), and type of study (randomized clinical trials). The search was carried out by combining the following Medical Subject Headings (MeSH) terms or keywords: “pelvic organ prolapse”, “POP”, “pelvic floor muscle training”, “pelvic floor muscle exercise”, “kegel exercise”, and “surgery”. In addition, the references of the selected articles were checked to identify studies that were not captured in the electronic searches. Only studies written in English or Spanish between 2011 and 2021 were considered. The search and selection of studies was carried out by two independent authors, with the differences that emerged being resolved by a third reviewer. Table 1 shows the searches performed with the limits in each database.

### 2.3. Inclusion and Exclusion Criteria

The inclusion criteria to be met by the studies of this review were: (1) the study had to be a randomized clinical trial (RCT); (2) the participants in each study had to be women with stage I–IV of POP according to the POP-Q system; (3) the intervention group received any type of PFMT program (including variations in the ways of teaching PFMT, in the type of contractions, and/or in the number of contractions); (4) the control group received any basic recommendations or performed an exercise program without PFMT; and (5) regarding the results, the study must report at least one of the following: symptoms associated with prolapse, prolapse stage (based on POP-Q data), quality of life, sexual function, or function of the pelvic floor.

The exclusion criteria were: (1) non-randomized controlled trials; (2) duplicated publications; (3) studies in which the participants used a pessary; and (4) studies that did not analyze or clearly describe any of the variables of interest.

### 2.4. Study Selection

In this stage, two authors screened articles by title and abstract in the consulted databases and independently applied the inclusion and exclusion criteria. Any article considered for potential inclusion by one of the authors was examined in detail. Disputes or differences of opinion were solved by a third reviewer.

### 2.5. Methodological Quality Assessment

Two authors independently used the PEDro scale to assess the quality of the included studies. This scale is made up of a total of 11 aspects, of which the first assesses external validity (and scores no points), and the remaining ten assess internal validity (the second to the tenth score points). Items are scored one point if answered “yes” and 0 if answered “no” because the criterion is not met. The sum of the value of the items concludes the value of the study’s quality, considering excellent quality in scores 9–10, good–high quality in scores 6–8, moderate quality in scores 4–5, and low quality in scores below 4 [15]. To reach a consensus, any discrepancies were resolved by discussion between the reviewers, consulting a third reviewer if necessary.

## 3. Results

**Figure 1:**
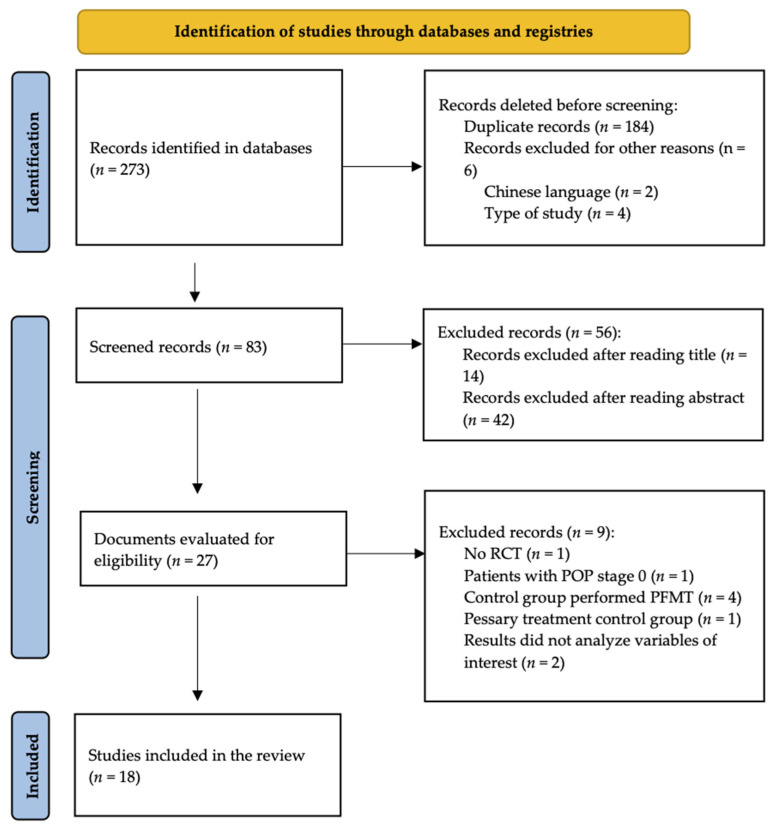
Study selection process.

Forty-five studies were identified in PubMed, 111 in SCOPUS, 60 in Cochrane, 20 in CINAHL, and 37 in PEDro as a result of the searches conducted in the different databases. Of these 273, 255 were excluded, and the remaining 18 studies were included in this review. The study selection process is summarized in Figure 1.

When evaluating methodological quality, four studies obtained a score of six, nine a score of seven, and two a score of eight on the PEDro scale, denoting good–high quality; whereas two studies obtained a score of four and one a score of five, denoting moderate quality (table of methodological quality according to the PEDro scale in Supplementary Table S1).

Ten studies evaluated the efficacy of PFMT as a treatment for POP patients, and eight studies evaluated the efficacy of PFMT as an adjunct treatment to POP surgery. Among the latter, in six studies, the patients underwent PFMT pre- and post-surgery, and in two studies, the patients underwent only pre-surgery. Table 2 shows the main characteristics of the studies.

### 3.1. POP-Q

Four studies [16,19,21,22] with 240 patients in the PFMT group and 221 in the control group reported a change in POP stage. One study [16] found significant improvement (*p* < 0.03 and effect size (ES) of 0.44) in anterior POP in the PFMT group, but no changes were found in posterior POP. Another study [22] reported significant improvement in both anterior and posterior POP in the PFMT group (*p* < 0.001 and *p* = 0.025, respectively). However, two studies [19,21] found no significant changes between the two groups.

### 3.2. Pelvic-Floor Distress Inventory (PFDI-20) and Subscales

Four studies conducted on two samples in different timelines [19,21,23,24], with 195 patients in the control group and 201 patients in the PFMT group, reported changes in urinary, intestinal, and pelvic symptoms using the PFDI-20 questionnaire and its subscales. In the study of Wiegersma, et al. [19], the PFMT group showed a significant improvement of 9.1 points greater than the control group (95% confidence interval (CI) −9.1(−15.4 to −2.8), *p* = 0.005). Within the subscales of the questionnaire, significant differences between groups were observed in the UDI-6 (95% CI −5.0(−8.6 to −1.4); (*p* = 0.007). At 24 months, with the same sample [24], the PFMT group improved significantly, with 12.2 points more than the control group (*p* < 0.001), and a significant difference was observed in favor of the PFMT group in the POPDI-6 and UDI-6 subscales (*p* < 0.001; *p* = 0.027 and *p* < 0.001, respectively). Due, et al. [21], did not find significant differences in the PDFI-20 or its subscales between the two groups at 3 or 6 months, although they found significant improvements in the PFMT group in the POPDI-6 subscale at 3 months (*p* = 0.001). At 12 months [23], they also found significant improvements (*p* = 0.01) in the POPDI-6 subscale in the PFMT group.

### 3.3. POP-SS

Two studies on the same sample [14,20] evaluated the changes in POP symptoms with the POP-SS questionnaire. In both studies, it was shown that PFMT significantly improved the symptoms associated with POP at 6, 12, and 24 months (*p* < 0.0001, *p* = 0.0053 and 95% CI −1.01(−1.7 to 0.33) *p* = 0.004, respectively).

### 3.4. Sexual Function (PISQ-12 and Specific Questionnaire)

Five studies [14,18,19,21,24] with 451 patients in the control group and 466 in the PMSC group reported changes in sexual function using the PISQ-12 questionnaire [14,18,19,21], while Braekken, et al. [18], used a specific and validated questionnaire for women with POP. No significant changes were found between the two groups in any of the studies.

### 3.5. Pelvic-Floor Function by Manual Assessment, EMG, or Manometry

Four studies [16,17,19,22] with 184 patients in the control group and 205 patients in the PFMT group reported information about pelvic-floor function (maximum voluntary contraction (MVC), strength, and/or endurance). Two studies [17,22] showed a significant increase in MVC, strength, and resistance of the pelvic floor in the PFMT group, and Alves, et al. [16], reported a significant increase in pelvic-floor strength in the intervention group both in manual assessment and in surface EMG (*p* = 0.001 and *p* = 0.003, respectively). The study of Wiegersma, et al. [19], concluded that the proportion of women in whom PF function either improved or deteriorated from baseline to follow-up was the same in both groups.

### 3.6. Quality of Life (P-QOL, ICIQ-VS, PFIQ-7, and Subscales)

Five studies [14,19,21,22,23] with 417 women in the control group and 428 in the intervention group evaluated the quality of life in patients with POP using different questionnaires. Wiegersma, et al. [19], reported improvement in quality of life in both groups, but no significant difference was found between them, which was in line with the results reported by Hagen, et al. [14]. Two studies [21,23] demonstrated significant improvements in CRAIQ-7 in the PFMT group compared with the control group at 6 and 12 months after the intervention (*p* = 0.037 and *p* = 0.04, respectively), although there were no significant differences at 3 months. One study [22] showed significant improvements in P-QOL in the PFMT group (*p* = 0.084).

### 3.7. Impression of Global Improvement in Patients and Change in Symptoms Reported by Patients

In the study of Due, et al. [21], with 53 patients in the control group and 56 in the intervention group, the impression of global improvement of the patients was reported by the PGI-I, in which a significant improvement was observed in the PFMT group at both 3 and 6 months after the intervention. Secondly, Wiegersma, et al. [19], reported changes in symptoms reported by the patients themselves (they asked if they were better, worse, or the same), in which 57% of the women in the intervention group reported an improvement in symptoms, 13% in the control group reported improvement, and 81% reported that their symptoms remained the same.

### 3.8. Surgery vs. Surgery + PFMT

Eight studies [25,26,27,28,29,30,31] reported on PFMT as a complementary treatment to surgery. The OPTIMAL clinical trial [25], with 188 women in the control group and 186 in the intervention group, showed that perioperative PFMT did not improve urinary symptoms at 6 months or the symptoms associated with POP at 2 years post-surgery, compared with surgical treatment alone. In a secondary report [29], no significant differences were found between the two groups in quality of life, sexual function, impression of improvement globally and by symptoms, pelvic-floor function, or body image at 6, 12, and 24 months after surgery. Five years after surgery [28], there were no significant differences in the symptoms associated with POP or until the time that anatomical failure of the POP occurred. In a more recent study [30] with 94 women, although improvement was reported in both groups at 40 and 90 days after surgery, no significant differences were found between groups in terms of urinary, intestinal, or pelvic symptoms, quality of life, sexual function, or pelvic-floor strength. Two recent studies on the same sample [27,31] demonstrated that pre-operative 22-week PFMT did not improve pelvic-floor strength, vaginal bulge sensation, anatomical change in POP, urinary and intestinal symptoms, or quality of life compared with the control group after 6 months of surgery. However, the study of Liang, et al. [26], with a sample of 90 women, showed that perioperative PFMT significantly improved urinary, intestinal, and pelvic symptoms 42 and 60 days after surgery (*p* <0.05). McClurg, et al. [32], also found significant differences between the two groups 12 months after the start of the treatment in favor of PFMT in terms of symptoms associated with POP (*p* = 0.006) and in quality of life (*p* = 0.004). Both groups reported similar results at 6 months with respect to the start of the treatment in terms of symptoms associated with POP (POP-SS), urinary symptoms (ICIQ-UI and ICIQ-BS), and quality of life (SF-12).

## 4. Discussion

Fifteen of the eighteen studies selected showed a good methodological quality score of six or more in PEDro. The findings of our review showed improvement in the symptoms associated with POP in women undergoing a PFMT protocol, including pelvic symptoms (pressure in the lower abdomen, feeling of heaviness in the pelvis, and feeling of a bulge in the vagina), urinary symptoms (stress urinary incontinence, urge urinary incontinence, sensation of incomplete bladder emptying, and pain when urinating), and bowel symptoms (constipation, fecal incontinence, gas incontinence, incomplete emptying of the bowel, pain when defecating, urgency, and bulging sensation in the anus). Improvements were also found in pelvic-floor function (MVC, strength, and endurance) and in quality of life. However, PFMT did not produce significant changes in sexual function, and the results regarding the change in POP stage were inconclusive. When PFMT was a complementary treatment to surgery, no significant improvements were observed in any of the analyzed variables.

It should be noted that the heterogeneity between the included trials made the interpretation of the findings more challenging. Furthermore, PFMT treatment protocols varied among studies, which could also cause more heterogeneity. The protocols differed in terms of the type of pelvic-floor contraction, since, in some studies, the authors asked the patients to perform MVC in a sustained manner [21,22,23,25,27,28,29,31,32], in other studies the patients were asked to perform fast contractions [14,20], and in others, patients were asked to perform a combination of both [17,26,30]. The number of total repetitions in each session ranged from 24 [22] to 180 [25], the frequency ranged from two times a week [16,30] to daily [14,17,18,20,25,26,27,28,29,31], and the total duration of the program ranged from 6 weeks [16] to 24 months [24]. Thus, it seems that PFMT could be useful regardless of the protocol used, although larger treatments obtained better results. In addition, not all studies indicated in which postures patients should perform PFMT at home, which would be interesting since the displacement of the pelvic floor during its contraction is not the same in supine, decubitus, or standing; the appearance of symptoms generally occurs when standing, and most of the activities of daily living are carried out in this position [33,34]. Based on this, future studies should consider performing PFMT also in standing position.

Our results coincide with those of previous studies. Li, et al. [8], based on 13 studies, showed that women who perform PFMT can significantly improve prolapse symptoms and muscle function. However, the results regarding the efficacy of PFMT as a complementary therapy to surgery were not conclusive due to the variability in the measurement methods. Zhang, et al. [35], analyzed five studies and indicated that no significant differences were found in PF function or POP stage when adding PFMT to surgery and when performing surgical treatment alone. Ge, et al. [36], based on 15 studies, showed that, compared with the control group, the PFMT group significantly improved the score on the POP-SS. These contradictory results could be explained by the inclusion of new studies in the review of Ge, et al. [36], or by the inclusion of participants who had undergone surgery, which would agree with our findings.

Two of the most significant symptoms in women with POP are vaginal heaviness and the feeling of a lump in the vagina. In women who underwent PFMT, we observed a notable improvement in pelvic, urinary, and intestinal symptoms. All of these were associated with a better quality of life, which is considered the most important treatment objective, since these symptoms are the main indication for surgery [8,36]. For this reason, and in absence of observed side effects, PFMT could be recommended for those POP patients with feeling of vaginal heaviness and related symptoms.

In all studies included in this review except two [19,24], the patients in the control group received lifestyle advice (avoid constipation, avoid being overweight, instruction in the Knack maneuver, management of chronic cough, smoking cessation, etc.); however, these studies show that the advice was not sufficient to observe improvement in symptoms and that it must be accompanied by supervised PFMT. In most of the studies, the patients performed PFMT at home in combination with sessions supervised by a physiotherapist, favoring adherence to the treatment. Kashyap, et al. [37], compared the efficacy of two PFMT protocols in a total of 140 women and with a 24-week follow-up. Both groups performed PFMT at home. Group A was instructed on how to perform PFMT through a brochure and only attended three follow-up sessions with the physiotherapist, while group B was instructed through an individual session, in which the physiotherapist checked how they performed the exercises, and attended six follow-up sessions. Given that significant differences were found in favor of group B in the mean POP-SS and PFIQ-7 scores at 6, 18, and 24 weeks of follow-up, it can be assumed that PFMT could be more effective when performed under supervision than when performed at home. Considering this, (1) future studies should compare PFMT with other active programs to assess which ones are more effective, and (2) any treatment based on PFMT must be performed under supervision of a specialized physiotherapist.

The support and stability of the pelvic organs are mainly carried out by the muscles of the levator ani and the pelvic ligaments. Pregnancy and vaginal delivery can cause weakness of the pelvic-floor muscles and are considered the most important risk factors for the development of POP. With an appropriate PFMT protocol, the pelvic floor can provide greater support to these organs, and the main objective of PFMT would be to improve the strength and endurance of the pelvic-floor musculature [8]. Our findings indicate that PFMT significantly increases pelvic-floor strength and endurance and that, consequently, there are improvements in POP symptoms [16,17,18,19,22]. Braekken, et al. [18], analyzed the changes produced in sexual function in women with POP who underwent PFMT. Although the majority of the women reported no changes in sexual function after 6 months of training, in the interview carried out in addition to the questionnaire, more women in the training group reported improvements compared with the control group (19 vs. 2); the main reasons given for this improvement were an increase in pelvic-floor awareness, strength, and control. Although questionnaires enable statistical comparisons, the study of sexual function using only questionnaires can be difficult, since they do not allow thorough study of this aspect and may not consider the evaluation that each individual makes as an improvement in sexual function.

When considering PFMT as a complementary treatment to surgery, eight studies [25,26,27,28,29,30,31,32] showed improvements in subjective POP, urinary, intestinal, and pelvic symptoms, quality of life, and pelvic-floor function in patients who only underwent surgery. In those who also performed PFMT in combination with surgery, no significant differences were found. The study of Liang, et al. [26], reported significant changes between both groups at 42 and 60 days post-surgery in terms of POP and urinary symptoms. However, since they did not collect the records of the PFMT participants, it cannot be fully confirmed that all patients adhered to the training. McClurg, et al. [32], reported significant changes between both groups in symptoms associated with POP and quality of life at 12 months after the intervention but not at 6 months, with adherence to treatment being similar in both groups (44% CG and PFMT group 50%). While the overall reduction in symptoms and anatomical changes of POP were expected after surgery, the improvement in pelvic-floor contraction was somewhat surprising and is not in line with the results of a recently published systematic review. This review concluded that there is no clear effect of POP surgery on pelvic-floor morphology or function [38], although the studies included in this review were heterogeneous and of low quality. It is also surprising that adding a PFMT protocol to surgical treatment does not produce significant differences in pelvic-floor function. One possible explanation for these findings is that the effect of surgery itself was so powerful that no difference could be found during the follow-up (although there is a significant deterioration in the success of the surgery over time [28]), or that the duration of 6 months of follow-up might not have been long enough to observe the additional benefits of PFMT. As we can see in the study of McClurg, et al. [32], no significant changes were observed in POP symptoms or quality of life between the two groups at 6 months post-surgery, although there were significant changes at 12 months in favor of the PFMT group. Another explanation could be that, in severe POP, it is difficult to perform PFMT and, when the prolapse is surgically reduced, the contraction and strength of the pelvic-floor increases. Long-term follow-up RCT are needed to clarify whether there are any effects of PFMT in addition to surgery, since only one study [28] assesses the effects 5 years after surgery.

Given the evidence that PFMT is effective in reducing symptoms of POP in stages I, II, and III, it seems essential that women with POP stages I to III are offered an evidence-based protocol of PFMT as first-choice treatment before surgery.

Our review had several limitations, mainly related to heterogeneity: the percentages of subjects with different stages of POP varied among studies; PFMT-protocol procedures varied among the included studies, in terms of frequency, intensity, and duration; the follow-up time also varied; and the variables to be measured and the measurement instruments were also heterogeneous. Moreover, limiting the language of the studies to English or Spanish may have reduced the results; and limiting the search to the last 10 years may have excluded older studies that could provide relevant information. Future reviews should include meta-analysis of data to provide more robust conclusions.

## 5. Conclusions

This review shows that PFMT program is effective for improving POP-associated pelvic, urinary, and intestinal symptoms and quality of life compared with controls. PFMT intervention also increases the strength and endurance of pelvic-floor musculature, but not the POP stage or sexual function. As an adjunct to prolapse surgery, the results of the included trials showed no benefit in adding PFMT to surgery compared with surgical treatment alone. Therefore, higher methodological-quality clinical trials with larger sample size and follow-up are needed to confirm or refute the results and conclusions.

## Figures and Tables

**Table 1:** Search strategies on each database.

Database	Search Strategy
PubMed	((“Pelvic Organ Prolapse” [Mesh] OR “POP”) AND (“pelvic floor muscle training” OR “pelvic floor muscle exercise” OR “kegel exercise”) AND (“surgery”))
SCOPUS	(TITLE-ABS ((“pelvic organ prolapse” OR “pop”)) AND TITLE-ABS ((“pelvic floor muscle training” OR “pelvic floor muscle exercise” OR “kegel exercise”))) AND TITLE-ABS (“surgery”))
Cochrane	(“Pelvic Organ Prolapse” OR “POP”) AND (“pelvic floor muscle training” OR “pelvic floor muscle exercise” OR “kegel exercise”) AND (“surgery”) in Record Title
Cinahl	AB (pelvic organ prolapse or pop) AND AB (pelvic floor muscle training or pelvic floor muscle exercise or kegel exercise) AND AB surgery
PEDro	Pelvic organ prolapse AND pelvic floor muscle training AND surgery

**Table 2:** Summary of selected studies.

Studies	Age		Population	N Patients		Applied Therapy		F-U
	C	I		C	I	C	I	
Alves et al. [16] 2015	65.67	66.11	Women POP I/II/III	10	18	Global exercise program without PFMT	Global exercise program (12 sessions) PFMT (home): 30 min × 2 times/wk, 6 wk	6 wk
Resende et al. [17] 2012	58.7	51.9	Women POP II	16	21	Lifestyle advice	PFMT (home): 3x8-12 rep MVC + 3 FC day, 3 months Lifestyle advice	3 months
Braekken et al. [18] 2015	48.3	49.4	Women POP I/II/III	50	59	Lifestyle advice	PFMT (supervised + home): 18 sessions + 3x8-12rep near MVC day, 6 months Lifestyle advice	6 months
Wiegersma et al. [19] 2014	64.0	64.5	Women POP I/II	142	145	Watchful waiting	PFMT with physiotherapist PFMT (home): 2–3 times/day, individual protocol, 3–5 times/wk, 3 months Lifestyle advice	3 months
Hagen et al. [20] 2014	57.50	56.20	Women POP I/II/III	222	224	Lifestyle advice	PFMT with physiotherapist 5 sessions PFMT (home): 3x10-50 FC a day, 16 wk Lifestyle advice	6 and 12 months
Due et al. [21] 2015	58	60	Women POP II/III	53	56	6 group sessions (Lifestyle advice)	6 group sessions + 6 PFMT with physiotherapist PFMT (home): 3x10 MVC maintained 10 sec, 5 days/wk, 12 wk Lifestyle advice	3 and 6 months
Hagen et al. [14] 2017	46.6	46.4	Women POP I/II/III	206	206	Lifestyle advice	5 sessions PFMT with physiotherapist PFMT (home): 3x10-50 FC a day, 16 wk Lifestyle advice	2 years
Stüpp et al. [22] 2011	58.12	52.95	Women POP II	16	21	Lifestyle advice	7 sessions PFMT with physiotherapist PFMT (home): 3x8-12 maintained contractions 6–10 sec, 12 wk Lifestyle advice	14 wk
Due et al. [23] 2016	-	-	Women POP II/III	43	40	6 group sessions (Lifestyle advice)	6 group sessions + 6 PFMT with physiotherapist PFMT (home): 3x10 MVC maintained 10 sec, 5 days/wk, 12 wk Lifestyle advice	12 months
Panman et al. [24] 2016	64.0	64.5	Women POP I/II	142	145	Watchful waiting	PFMT with physiotherapist PFMT (home): 2–3 times/day, individual protocol, 3–5 times/wk, 2 years	12 months 2 years
Barber et al. [25] 2014	56.9	57.5	Women POP II/III/IV	188	186	Surgery + routine perioperative care	Surgery + PFMT (supervised + home) PRE: 1 session, POST: 4 sessions HOME: individualized protocol 3x45-60 rep 1–10 sec contraction, at 3 months 15 contractions daily max duration, 24 months	6 months and 2 years
Liang et al. [26] 2019	63.3	61.6	-	43	47	Surgery + Lifestyle advice	Surgery + PFMT (supervised + home): PRE: 1 session, POST: 3 sessions HOME: 2–3x15–30 min 10 sec contraction-10 sec rest + FC daily, 60 days	42 and 60 days
Nyhus et al. [27] 2020	60.6	60.1	Women POP ≥ II	76	75	Surgery	Surgery + PFMT PRE (home): 3x8-12 maintained contractions 6–8 sec daily Group option 1 day/wk 22 wk	6 months
Jelovsek et al. [28] 2018	57.4	57	Women POP II/III/IV	144	141	Surgery + routine pre-operative care	Surgery + PFMT (supervised + home) PRE: 1 session, POST: 4 sessions HOME: individualized protocol 3x45-60 rep 1–10 sec contraction, at 3 months 15 contractions daily max duration, 24 months	5 years
Weidner et al. [29] 2017	56.9	57.5	Women POP II/III/IV	188	186	Surgery + routine pre-operative care	Surgery + PFMT (supervised + home) PRE: 1 session, POST: 4 sessions HOME: individualized protocol 3x45-60 rep 1–10 sec contraction, at 3 months 15 contractions daily max duration, 24 months	6 and 12 months 2 years
Duarte et al. [30] 2020	-	-	Women POP II/III/IV	46	48	Surgery	Surgery + PFMT (supervised + home): PRE: 2 times/wk, 2 wk, POST: 7 sessions HOME: 3 days/wk, 4x10 MVC 7 sec contraction 7 sec rest + 5 FC	40 and 90 days
Mathew et al. [31] 2021	60.6	60.1	Women POP ≥ II	76	75	Surgery	Surgery + PFMT (home): 3x8-12 maintained contractions 6–8 sec daily. Group option 1 day/wk 22 wk	6 months
McClurg et al. [32] 2013	60	60	Women POP I/II/III	29	28	Surgery + routine pre-operative care	Surgery + PFMT: PRE (home): 3x10 MVC held up to 10 sec and 4 sec rest POST (supervised + home): 5 sessions/wk, 12 wk, individualized protocol with BFB, EE and exercise balls if necessary	6 and 12 months

C: control group; I: intervention group; POP: pelvic organ prolapse; F-U: Follow-up; PFMT: pelvic-floor muscle training; min: minutes; rep: repetitions; wk: week; MVC: maximum voluntary contraction; FC: fast contractions; sec: seconds; PRE: pre-surgery; POST: post-surgery; max: maximum; BFB: biofeedback; EE: electrostimulation.

## Data Availability

Not applicable.

## Supplementary Materials

The following supporting information can be downloaded at: https://www.mdpi.com/article/10.3390/jpm12050806/s1, Supplementary Table S1: Assessment of methodological quality with PEDro scale.
