# Supplementary material for: Effects of Pelvic-Floor Muscle Training in Patients with Pelvic Organ Prolapse Approached with Surgery vs. Conservative Treatment: A Systematic Review
Source: J Pers Med. 2022 May 17;12(5):806. doi: 10.3390/jpm12050806 (PMC9142907; doi:10.3390/jpm12050806)
Supplement: Supplementary file 1 [file jpm-12-00806-s001.zip › jpm-1668743-supplementary.pdf]

Supplementary Table S1. Assessment of methodological quality with PEDro scale

Criterion ***Study***	*Eligibility criteria*	*Random allocation*	*Concealed allocation*	*Baseline comparability*	*Blind subjects*	*Blind therapists*	*Blind assessors*	*Adequate follow-up*	*Intention-to-treat analysis*	*Between-group comparisons*	*Point estimates and variability*	*Total score (over 10) **
Alves 2015	Yes	Yes	Yes	Yes	No	No	Yes	No	No	Yes	Yes	6
Resende 2012	No	Yes	No	Yes	No	No	Yes	Yes	No	Yes	Yes	6
Braekken 2015	Yes	Yes	Yes	Yes	No	No	No	Yes	Yes	Yes	Yes	7
Wiegersma 2014	Yes	Yes	Yes	Yes	No	No	No	Yes	Yes	Yes	Yes	7
Hagen 2014	Yes	Yes	Yes	Yes	No	No	Yes	No	Yes	Yes	Yes	7
Due 2015	Yes	Yes	Yes	Yes	No	No	Yes	No	Yes	Yes	Yes	7
Hagen 2017	Yes	Yes	Yes	Yes	No	No	No	No	Yes	Yes	Yes	6
Stüpp 2011	Yes	Yes	No	Yes	No	No	Yes	No	No	Yes	Yes	5
Due 2016	Yes	Yes	No	No	No	No	Yes	No	No	Yes	Yes	4
Panman 2016	Yes	Yes	Yes	Yes	No	No	Yes	Yes	Yes	Yes	Yes	8
Barber 2014	Yes	Yes	Yes	Yes	No	No	No	Yes	No	Yes	Yes	6
Liang 2019	Yes	Yes	No	Yes	No	No	Yes	Yes	Yes	Yes	Yes	7
Nyhus 2020	Yes	Yes	No	Yes	No	No	Yes	Yes	Yes	Yes	Yes	7
Jelovsek 2018	Yes	Yes	No	Yes	No	No	Yes	Yes	Yes	Yes	Yes	7
Weidner 2017	Yes	Yes	Yes	Yes	No	No	No	No	No	Yes	No	4
Duarte 2020	Yes	Yes	Yes	Yes	No	No	Yes	Yes	Yes	Yes	Yes	8
Mathew 2021	Yes	Yes	No	Yes	Yes	No	Yes	Yes	Yes	Yes	Yes	7
McClurg 2013	No	Yes	Yes	Yes	No	No	Yes	No	Yes	Yes	Yes	7

**Note: Eligibility criteria item does not contribute to total score.*

Notes on administration of the PEDro scale:

All criteria. Points are only awarded when a criterion is clearly satisfied. If on a literal reading of the trial report it is possible that a criterion was not satisfied, a point should not be awarded for that criterion.

Criterion 1. This criterion is satisfied if the report describes the source of subjects and a list of criteria used to determine who was eligible to participate in the study.

Criterion 2. A study is considered to have used random allocation if the report states that allocation was random. The precise method of randomisation need not be specified. Procedures such as coin-tossing and dice-rolling should be considered random. Quasi-randomisation allocation procedures such as allocation by hospital record number or birth date, or alternation, do not satisfy this criterion.

Criterion 3. *Concealed allocation* means that the person who determined if a subject was eligible for inclusion in the trial was unaware, when this decision was made, of which group the subject would be allocated to. A point is awarded for this criteria, even if it is not stated that allocation was concealed, when the report states that allocation was by sealed opaque envelopes or that allocation involved contacting the holder of the allocation schedule who was "off-site".

Criterion 4. At a minimum, in studies of therapeutic interventions, the report must describe at least one measure of the severity of the condition being treated and at least one (different) key outcome measure at baseline. The rater must be satisfied that the groups' outcomes would not be expected to differ, on the basis of baseline differences in prognostic variables alone, by a clinically significant amount. This criterion is satisfied even if only baseline data of study completers are presented.

Criteria 4, 7-11. *Key outcomes* are those outcomes which provide the primary measure of the effectiveness (or lack of effectiveness) of the therapy. In most studies, more than one variable is used as an outcome measure.

Criterion 5-7. *Blinding* means the person in question (subject, therapist or assessor) did not know which group the subject had been allocated to. In addition, subjects and therapists are only considered to be "blind" if it could be expected that they would have been unable to distinguish between the treatments applied to different groups. In trials in which key outcomes are self-reported (eg, visual analogue scale, pain diary), the assessor is considered to be blind if the subject was blind.

Criterion 8. This criterion is only satisfied if the report explicitly states both the number of subjects initially allocated to groups and the number of subjects from whom key outcome measures were obtained. In trials in which outcomes are measured at several points in time, a key outcome must have been measured in more than 85% of subjects at one of those points in time.

Criterion 9. An *intention to treat* analysis means that, where subjects did not receive treatment (or the control condition) as allocated, and where measures of outcomes were

available, the analysis was performed as if subjects received the treatment (or control condition) they were allocated to. This criterion is satisfied, even if there is no mention of analysis by intention to treat, if the report explicitly states that all subjects received treatment or control conditions as allocated.

Criterion 10. A *between-group* statistical comparison involves statistical comparison of one group with another. Depending on the design of the study, this may involve comparison of two or more treatments, or comparison of treatment with a control condition. The analysis may be a simple comparison of outcomes measured after the treatment was administered, or a comparison of the change in one group with the change in another (when a factorial analysis of variance has been used to analyse the data, the latter is often reported as a group × time interaction). The comparison may be in the form hypothesis testing (which provides a "p" value, describing the probability that the groups differed only by chance) or in the form of an estimate (for example, the mean or median difference, or a difference in proportions, or number needed to treat, or a relative risk or hazard ratio) and its confidence interval.

Criterion 11. A *point measure* is a measure of the size of the treatment effect. The treatment effect may be described as a difference in group outcomes, or as the outcome in (each of) all groups. *Measures of variability* include standard deviations, standard errors, confidence intervals, interquartile ranges (or other quantile ranges), and ranges. Point measures and/or measures of variability may be provided graphically (for example, SDs may be given as error bars in a Figure) as long as it is clear what is being graphed (for example, as long as it is clear whether error bars represent SDs or SEs). Where outcomes are categorical, this criterion is considered to have been met if the number of subjects in each category is given for each group.
